# Research on Adoption Behavior and Influencing Factors of Intelligent Pension Services for Elderly in Shanghai

**DOI:** 10.3389/fgene.2022.905887

**Published:** 2022-06-15

**Authors:** Juan Luo, Lingqi Meng

**Affiliations:** ^1^ School of Social Development and Public Policy, Fudan University, Shanghai, China; ^2^ Shanghai University of Engineering Science, Shanghai, China

**Keywords:** artificial intelligence, intelligent pension, pension model, influencing factors, home care

## Abstract

With the rapid development of artificial intelligence and Internet-of-Things technology, the traditional pension service mode has changed, and intelligent pension services have become a new direction of development. Descriptive statistical analysis is conducted on the supply status and demand of intelligent pension services. It is believed that the current intelligent pension services are still in the initial stage of development, and the contradiction between supply and demand is prominent. The demand for intelligent pension of the elderly is high, but the user acceptance and satisfaction are not high. On this basis, variables were selected from individual characteristics, family situation, economic status, education level, living conditions, and other indicators for multivariate unconditional logistic regression analysis. It was found that the adoption behavior of intelligent pension service users was most significantly affected by age, number of children, living conditions, service cost, service docking channel, and equipment operation difficulty. Based on the conclusion, this article puts forward some suggestions such as taking the government as the center to realize the multi-governance of intelligent pension services, improving the supply of intelligent pension service-related facilities guided by demand, optimizing the service mode based on the platform to realize dynamic combination, and taking talents as the core to promote the high-quality development of intelligent pension services.

## 1 Research Review and Questions

Population aging has become an inevitable trend of social development. The National Office for Aging released a research report on the prediction of population aging trend in China, and it is expected that China will enter the accelerated aging stage from 2021 and the severe aging stage from 2051, and the aging trend will gradually increase. The modernization of society has spawned profound social changes. With the vigorous development of Internet-of-Things technology and big data, artificial intelligence has gradually been applied to the field of pension services and has become an important driving force for the transformation of traditional pension models. The issuance of Opinions on Promoting the Development of Pension Service by the General Office of the State Council in 2019 and the deployment requirements of the Outline of Healthy China 2030 in China have clarified the important role of improving the quality of pension service development in the whole process of social development. In 2017, the Ministry of Industry and Information Technology, the Ministry of Civil Affairs, and the Health and Family Planning Commission jointly issued “Smart Health Care Industry Development Action Plan (2017–2020),” raising the development of the smart and healthy pension industry to the national strategy. As an international metropolis, Shanghai has relatively high financial support capacity. Perfect network infrastructure construction has become a favorable condition for carrying out community intelligent pension services. The development of preliminary pilot work has also accumulated experience for the later operation of intelligent pension services. Shanghai has certain typicality and representativeness in the development of home intelligent pension services.

Combined with the current domestic research, many scholars have defined the concept and explained the connotation of intelligent pension. Most of them believe that intelligent pension is a new pension service model that relies on Internet technology and uses terminal equipment and data platform to provide effective pension services (Zhu and Tang, 2020). Intelligent pension services focus on the integration of pension and medical resources, and top-level design has strong comprehensive requirements (Wu, 2019). Compared with the traditional pension service mode, intelligent pension has the characteristics of high efficiency, which can maximize the action ability of the elderly, expand the rights of the elderly, and effectively reduce the pressure of social support (Jain et al., 2021). Relevant research has been conducted on the influencing factors of the demand for intelligent pension services. From the perspective of the elderly’s own life security needs, Bai Mei et al. (2015) found through a survey of Wuhan that the elderly at different ages will have different willingness to adopt smart pension services due to the influence of factors such as living conditions, economic conditions, and physical conditions. Mao and Li (2015) proposed eight influencing factors based on the UTAUT model, including performance expectation, convenience condition, perceived security, and perceived trust. The study found that convenience condition was the key factor affecting the demand for intelligent pension services. Gao et al. (2019), through the empirical research on the development of smart pension in Bincheng District, put forward the influencing factors of smart pension service demand such as cultural education, children’s situation, and their own quality. From the perspective of service providers, Zhang Lei (2017) believes that the future market development prospect of smart pension is optimistic, but there are many problems in technical means and standard formulation (Zhang and Han, 2017). These factors have become an important factor restricting the promotion of the smart pension mode.

At the same time, some scholars believe that the behavior choice of intelligent pension service users is also affected by external risk factors. The conflict of interest between the supply and demand of smart pension services is the fundamental reason for the difficulties in its popularization and promotion (Zhang and Li, 2019). At present, the overall problem of smart pension services in China is the shortage of total supply and the structural contradiction between supply and demand, which forms the urgency of the supply-side structural reform of smart pension services (Liao, 2019). The contradiction between supply and demand structures of intelligent pension services is manifested in the mismatch between the supply and demand of products and services and the imbalance between regional supply and demand. In terms of services and products, China’s intelligent pension services are less involved and do not match the diversified and personalized needs of pension services. Therefore, the research on the demand side of the intelligent pension service is the basis of supply side structural reform and has important research value and significance.

On the whole, the current research on the demand and influencing factors of the intelligent pension service has made some achievements, but the empirical research on the demand side is still less, especially the lack of relevant research on the influence of subsequent elderly users’ adoption behavior. On the basis of relatively fully grasping the influencing factors of intelligent pension service demand, it is also necessary to conduct more in-depth research on the influencing factors that determine the final adoption behavior of user groups so as to better transform demand into practical application. The implementation of smart pension services in Shanghai is relatively considerable, and it is representative and exemplary in China. On the basis of household registration, salary level, education level, and other existing characteristics, this article incorporated children, living conditions, and other factors into the analysis variables and explored the relevant situation of the users’ adoption behavior of smart pension services in Shanghai in many aspects, aiming to find an effective path for the development of smart pension services.

## 2 Data Sources of Intelligent Pension

### 2.1 Defining the Scope of Investigation

The survey covers 15 districts in Shanghai, and Chongming District is not covered due to its remote geographical location. The selected streets in each district are mainly “smart healthcare demonstration street” and “smart pension community” in Shanghai.

### 2.2 Subjects of the Survey

The subjects of this survey were the elderly population over 60 years old in Shanghai.

### 2.3 Survey Methods and Tools

In 2019, the total number of elderly people over 60 years of age in the Shanghai Bureau of Statistics was 518.12 million. According to the proportion of 0.01%, a total of 518 elderly people were to be investigated. In order to ensure the quality of the questionnaire and eliminate invalid questionnaires, the number of respondents is now increased to 550. In a self-designed questionnaire, the questionnaire content included four main parameters: demographic data, intelligent pension service supply status, intelligent pension service supply satisfaction, and intelligent pension service demand status. Demographic data include gender, age, educational level, income, number of children, and living conditions; the supply status of intelligent pension services includes the cognition of users of intelligent pension services, the equipment of intelligent pension services, the use of intelligent pension services, and the sources of information acquisition of intelligent pension services. The satisfaction degree of intelligent pension service includes the satisfaction degree and influencing factors of users to the existing intelligent pension service; the demand for intelligent pension services includes user preference for the types of intelligent pension services, the degree of demand for intelligent pension services, and influencing factors. A total of 550 questionnaires were distributed, and 531 valid questionnaires were recovered, with an effective rate of 96.5%.

### 2.4 Research on Demand and Influencing Factors of the Intelligent Pension Service

The collected questionnaires were collated and coded, and the relevant data were analyzed by SPSS statistical software. Demographic data and the supply and demand status of intelligent pension services were analyzed by descriptive statistics, and the influencing factors of user adoption behavior of intelligent pension services were studied by the multivariate unconditional logistic regression analysis.

## 3 Supply, Demand, and Influencing Factors of the Intelligent Pension Service

### 3.1 Descriptive Statistical Analysis of Demographic Data

In this survey, the proportion of females in intelligent pension service users is slightly higher than that of males, and the age distribution is mainly young elderly (60–69 years old), accounting for about 50.3%. The educational level of the survey users is generally concentrated in the primary and junior high school stages. Combined with the analysis of the background of the times, most of the elderly groups were born before the 1950s. Affected by social and economic factors, the cultural level is generally low. The majority of children are less than three, and the pressure to support the elderly is high. About 43.4% of the intelligent pension service users’ income is more than 5,000 yuan; 67.3% of the intelligent pension service users’ funds mainly rely on pension, and 24.8% are given by their children. The overall income of the elderly group is relatively considerable, but the access to security funds is relatively single, and there are still some elderly people in poverty. In the choice of living style, 48.4% of the elderly live with their lovers, 28.2% of the elderly live with their children, and the proportion of the elderly living alone is about 5.1%. Table 1 shows some basic descriptive data that have been analyzed.

**TABLE 1:** Descriptive statistics of demographic characteristics.

Variable	Classification	Number of people (n)	Percentage (%)
Sexuality	Males	232	43.7
	Females	299	56.3
Age	60–69 years	265	50.3
	70–79 years	216	41.0
	80–89 years	44	8.3
	Above 90 years old	2	0.4
Educational level	Uneducated	17	3.2
	Primary school	129	24.3
	Junior high school	163	30.7
	Technical secondary school	95	17.9
	High school	66	12.4
	Junior college	44	8.3
	Undergraduate	17	3.2
Number of children	Five or more	14	2.6
	Four	26	4.9
	Three	38	7.2
	Two	151	28.4
	One	298	56.1
	Nil	4	0.8
Income	Less than 2,000 yuan	19	3.6
	2,001–3,000 yuan	43	8.1
	3,001–4,000 yuan	109	20.5
	4,001–5,000 yuan	130	24.5
	More than 5,000 yuan	230	43.4
Revenue sources	Income from work	20	2.90
	Retirement pay	472	67.30
	Children give	174	24.80
	Government subsidies	34	4.90
	Other	1	0.10
Living condition	Individual living alone	27	5.1
	With lover	257	48.4
	With children	150	28.2
	With lover and children	95	17.9
	With the elderly	2	0.4

### 3.2 Status Quo of Smart Elderly Service Supply

The survey results showed that the elderly population has relatively low understanding of smart elderly care services. Among them, 59.3% of the elderly do not know much about smart elderly care services, 10.9% have never heard of smart elderly care services, and 23.4% of the elderly have a basic understanding. The elderly who are relatively and very knowledgeable about intelligent elderly care services account for only 5.8% and 0.4%, respectively. In order to more intuitively show the understanding of the elderly on intelligent pension services, we presented this in Table 2.

**TABLE 2:** Degree of understanding of intelligent elderly care services.

Understanding of existing smart elderly care services	Frequency	Percentage (%)	Effective percentage (%)	Cumulative percentage (%)
Know very well	2	0.4	0.4	0.4
Better understanding	31	5.8	5.8	6.2
Basically understood	124	23.4	23.4	29.6
Not well understood	315	59.3	59.3	88.9
Never heard	58	10.9	10.9	99.8

At present, the overall satisfaction of smart elderly care service users is not high. The proportion of relatively dissatisfied and very dissatisfied users is as high as 56.7%, the generally satisfied users account for 16.2%, and the very satisfied and relatively satisfied users account for only 11.3%. It can be seen that the development of intelligent elderly care services is still in the preliminary stage of development. Users lack relevant cognition and have a low understanding of community intelligent elderly care, which affects the further promotion and application of community intelligent elderly care service, and there is still much room for improvement and development. The satisfaction of the elderly group to intelligent elderly care service is illustrated in Table 3.

**TABLE 3:** Satisfaction with smart elderly care services.

Satisfaction with existing smart elderly care services	Frequency	Percentage (%)	Effective percentage (%)	Cumulative percentage (%)
Very satisfied	54	10.2	10.2	10.2
Quite satisfied	6	1.1	1.1	11.3
General	86	16.2	16.2	27.5
Less satisfied	256	48.2	48.2	75.7
Very dissatisfied	45	8.5	8.5	84.2

In terms of the factors affecting the satisfaction of smart elderly care services, users generally believe that the types of services and service personnel are the main factors affecting satisfaction. Among them, 28.6% of the elderly believe that the current smart elderly services provide fewer types of services, and 22.4% of the elderly believe that there are fewer professional service personnel, and second, service charges and service timeliness are also important aspects that affect user satisfaction. In order to make the data results more intuitive, we showed the factors influencing the satisfaction of intelligent pension services in Table 4.

**TABLE 4:** Factors affecting satisfaction with smart elderly care services (unit: person).

Factors influencing satisfaction of smart elderly care services	Number of response cases	Percentage (%)	Percentage of cases (%)
Unreasonable fees	115	16.50	26.20
Poor service timeliness	104	14.90	23.70
Cannot provide customized services	99	14.20	22.60
Fewer professional service staff	156	22.40	35.50
Fewer professional service staff	199	28.60	45.30
Other	23	3.30	5.20

### 3.3 Description and Analysis of User Needs and Adoption Behavior of Intelligent Elderly Care Services

#### 3.3.1 Analysis of Needs and Preferences of Smart Elderly Care Users

Through statistical analysis, it is found that the needs of the elderly for intelligent elderly care services are mostly concentrated in the two aspects of life care and medical care, followed by spiritual comfort, and most of them have moderate needs in cultural education and fitness. The demand for legal services is relatively low. Combining with Maslow’s hierarchy of needs theory, the current relative basics of the life needs of the elderly are mostly related to the physical and social needs of the fetish and spiritual levels, and the degree of self-realization needs is relatively weak. Table 5 shows the demand preference analysis of the elderly for intelligent elderly care services.

**TABLE 5:** Analysis of demand preference for smart elderly care services (unit: person).

Demand level	Life care	Medical insurance	Spiritual comfort	Cultural education	Physical fitness	Legal service
Not needed at all	44 (8.3%)	8 (1.5%)	64 (12.1%)	75 (14.1%)	79 (14.9%)	137 (25.8%)
Not needed	40 (7.5%)	21 (4.0%)	83 (15.6%)	151 (28.4%)	137 (25.8%)	155 (29.2%)
General	90 (16.9%)	74 (13.9%)	120 (22.6%)	130 (24.5%)	146 (27.5%)	119 (22.4%)
Need	126 (23.7%)	128 (24.1%)	132 (24.9%)	106 (20.0%)	93 (17.5%)	71 (13.4%)
Very necessary	231 (43.5%)	300 (56.5%)	132 (24.9%)	69 (13.0%)	75 (14.1%)	49 (9.2%)

#### 3.3.2 Analysis of the Adoption Behavior of Smart Elderly Service Users

Contrary to the distribution of demand level surveys, the elderly population generally has a higher level of demand for smart elderly care services but seldom adopts smart elderly care services. Among them, 41.8% of elderly users are equipped with less smart elderly care service equipment, and 23.5% of the elderly population are equipped with average smart elderly care equipment. There are still 28.6% of the elderly who are basically not equipped with any smart elderly care equipment. The users with much or smarter elderly care equipment are around 0.8% and 5.1%, respectively. The frequency analysis of providing intelligent elderly care service facilities is presented in Table 6.

**TABLE 6:** Provision of smart elderly service facilities.

Provision of intelligent elderly service facilities	Frequency	Percentage (%)	Effective percentage (%)	Cumulative percentage (%)
Much	4	0.8	0.8	0.8
More	27	5.1	5.1	5.8
General	125	23.5	23.5	29.4
A bit less	222	41.8	41.8	71.2
Basically no	152	28.6	28.6	99.8

The types of smart elderly care equipment currently equipped by elderly users are relatively basic; 27.4% are smart home appliances, 16.2% are smart phone watches, and the proportion of other smart elderly care equipment is below 10%. Mental health self-service equipment is particularly weak. There are 2.9%. From this point of view, the current devices adopted by smart elderly care users are mostly concentrated on the auxiliary devices of daily life, and the adoption behavior in medical health and psychological services is relatively lacking. The current situation of elderly people equipped with intelligent elderly care service equipment is shown in Table 7.

**TABLE 7:** Equipped with smart elderly service equipment.

Already equipped with intelligent elderly care equipment	Number of response cases	Percentage (%)	Percentage of cases (%)
Intelligent walkway	45	5.4	9.9
Health self-examination cabin	35	4.2	7.7
Self-help physical examination apparatus	60	7.2	13.2
Physical fitness monitoring station	74	8.8	16.3
Mental health self-help instrument	24	2.9	5.3
Intelligent health monitor	74	8.8	16.3
Smartphone watches	136	16.2	30
Intelligence appliance	230	27.4	50.7
Guided robot	41	4.9	9
Intelligent fitness equipment	38	4.5	8.4
Other	82	9.8	18.1

## 4 Logistic Regression Analysis of Factors Influencing the Adoption Behavior of Smart Elderly Service Users

### 4.1 Variable Assignment

Seven factors, including gender, age, education level, income, and living conditions, are included as independent variables. The dependent variable is “whether to use smart pension service” (yes = 1 and no = 0), and use multi-factor unconditional logistic analysis to explore the factors affecting the demand for smart elderly services. For the logistic regression analysis of the factors influencing the adoption behavior of intelligent elderly care service users, we have allocated the relevant variables, as shown in Table 8.

**TABLE 8:** Assignment of related variables.

Relevant factor	Option	Assignment	Relevant factor	Option	Assignment
Gender	Male	1	Living situation	Individual living alone	1
	Female	2		With lover	2
Age	60–69 years	1		With children	3
	70–79 years	2		With lover and children	4
	80–89 years	3		With the elderly	5
	Over 90 years old	4		Individual living alone	6
Education	Uneducated	1	Number of children	Five or more	1
	Primary school	2		Four	2
	Junior high school	3		Three	3
	Technical secondary school	4		Two	4
	High school	5		One	5
	Junior college	6		Nil	6
	Undergraduate	7	Service provision conditions	Expense cost	1
Income situation	Below 2,000 yuan	1		Information security	2
	2,001–3,000 yuan	2		Service docking by the community	3
	3,001–4,000 yuan	3		Combined with traditional service methods	4
	4,001–5,000 yuan	4		Simple equipment operation	5
	Above 5,000 yuan	5			

### 4.2 Result Analysis of Factors Affecting the Adoption Behavior of Smart Elderly Service Users

The influencing factors of adoption behavior of intelligent home-care users in Shanghai are not the same. This article used the multivariate unconditional logistic regression method to analyze. The adoption behavior of intelligent pension users was set as “Y”, independent variable X = (x1, x2, ... , x7), “P” was the response probability of the model, and the corresponding logistic regression model is as follows:

$$Y_{i}=\ln\left(\frac{p_{i}}{p_{m}}\right)=\beta_{0}+\sum_{j=1}^{n}\beta_{j}X_{j}\left(j=1,2,3,4,5,6,7\right).$$

The survey data showed that the correlation of age, number of children, living conditions, service cost, service docking channels, equipment operation difficulty, and other factors are less than 0.05, that is, there is an obvious correlation between each factor and adoption behavior. In other words, it has a significant impact on the adoption behavior of intelligent elderly service users.

Among the demographic characteristics, factors such as age, number of children, and living conditions have a significant impact on the demand for intelligent elderly care services. Among them, the elderly aged 60–79 have a significant positive impact on the demand for smart elderly care, that is, the older the elderly, the higher is the demand for smart elderly care services. Analysis of the reasons shows that as age increases, whether the elderly are in action, there will be more and more needs in terms of medical and health care, life care, and meal services. However, for the elderly over 80 years old, age has no effect on their demand for intelligent elderly care services. The only child is also an important factor affecting the demand for intelligent elderly care services. In order to reduce the pressure of supporting children, the elderly will prefer to choose intelligent elderly care services in order to reduce the pressure of care and supervision in daily life. The living environment is also an influencing factor in the demand for intelligent elderly care services. Elderly people living alone generally lack the physical and psychological care provided by family members. They will encounter more risk problems in their daily life, and the additional risks brought by age increase are increasing. The demand for smart elderly care services is also increasing.

In terms of service provision needs, the elderly care about whether the use of intelligent elderly care services is suitable for the elderly. The difficulty of equipment operation and the connection channels of the services will affect the adoption behavior of the elderly. If the service provision is suitable for the elderly, the elderly user groups are generally willing to accept intelligent elderly care services. This may be because with the increase in age and the influence of the educational background, the elderly groups are generally weak in accepting and adapting to new things. Insufficient age-appropriateness of intelligent elderly care equipment will cause the elderly to face various problems during their use, which will increase the pressure on the provision of elderly care services. Table 9 shows the results of the logistic regression analysis of factors influencing the adoption behavior of intelligent elderly service users.

**TABLE 9:** Logistic regression analysis results of factors influencing the adoption behavior of smart elderly service users.

Variable		B	S.E	Wals	*p* value	Exp (B)
Age	60–69 years	0.759	0.216	12.319	0.000	0.468
	70–79 years	1.433	0.442	10.498	0.001	0.239
Only child		1.308	0.623	4.412	0.036	0.27
Individual living alone		1.2	0.351	11.727	0.001	3.321
Expense cost		−0.886	0.267	11.016	0.001	0.412
Service docking by the community		0.762	0.2	14.497	0.000	0.467
Simple equipment operation		1.474	0.237	38.62	0.000	0.229

## 5 Factors and Problems Restricting the Development of Intelligent Elderly Care Services

### 5.1 Insufficient Motivation for the Development of Smart Elderly Care Services, Lack of Guidance, and Supervision Mechanisms

The target of intelligent elderly care services is the elderly, and the intelligent service methods are still relatively unfamiliar to them. At present, the popularity of intelligent elderly care services is relatively low, largely because the elderly have a low awareness of new elderly care methods and methods. Through the survey, it is found that the elderly population has a general understanding of smart elderly care services. Among them, 59.3% of the elderly do not know much about smart elderly care services, 10.9% have never heard of smart elderly care services, and 23.4% of the elderly have basic knowledge. Only 5.8% and 0.4% of the elderly have knowledge and knowledge of intelligent elderly care services, respectively, and the information source channels are also concentrated on community bulletin boards, the Internet, and TV. It can be seen that the government and society have relatively weak publicity for intelligent elderly care services, and the relatively single channel for the elderly to obtain information makes it more difficult to promote intelligent elderly care services. At the same time, as a new type of elderly care service, the government has not established complete legal safeguards. There are still many system loopholes in information security, ethics, and quality supervision. When the elderly are exposed to new things, they will be more cautious. In the absence of the corresponding supervision measures and system protection, the acceptance of the elderly will be greatly affected.

### 5.2 Construction of the Intelligent Elderly Care Service Platform Lacks Linkage, and the Service Supply Application Is Poor for the Elderly

The construction of intelligent elderly care services is still in the initial stage of development, and the items and contents of the service supply are still very basic, resulting in inadequate access to and processing of health data, and a complete sharing mechanism has not yet formed. Each service item is independent and independent of each other. The linkage is poor. If elderly users want to adopt smart elderly care services, they need to be equipped with a variety of smart devices. The cumbersome use process will seriously affect the elderly’s sense of use. In terms of the technical design of smart elderly care services, the complicated operation steps of related equipment, small screens, small buttons, and unclear voices will also affect the suitability of smart elderly care services. In addition, children spend less time with children and cannot help the elderly at any time. There remain problems with the use of equipment. Therefore, the poor adaptability of smart elderly care services will seriously reduce the desire to use by the elderly.

### 5.3 Supply and Demand of Smart Elderly Care Services Are Not Matched, and the Product Types Are Severely Homogenized

In the questionnaire survey, it was found that the elderly equipped with more smart products and devices are smart home appliances and smart phone watches, which are less equipped with equipment similar to self-service physical examination equipment and mental health self-service equipment. In the needs survey, it was found that the needs and preferences of the elderly group were concentrated in life care, medical care, and mental health. In related surveys that affect the satisfaction of elderly care services, most people think that the lack of service types is an important factor. From this point of view, the current supply and demand of smart elderly care services are relatively low, and the smart elderly care equipment currently provided cannot meet the diverse needs of the elderly for smart elderly care services. At the same time, the current smart elderly care lacks affordable customized services for elderly people of different ages, different living habits, and different cognitive abilities. The survey found that more than half of the elderly believe that the cost of smart elderly care services should be borne by most government subsidies; the rest is paid by the individual. Therefore, the price factor is an obstacle to the promotion of intelligent elderly care services.

## 6 Countermeasures and Suggestions for the Development of Intelligent Elderly Care Services

### 6.1 Realizing Multiple and Co-Governance of Intelligent Elderly Care Services With the Government as the Center

The supply of smart elderly care services involves multiple responsible entities. In order to eliminate the fragmentation problems in the supply of smart elderly care, it is necessary to unite multiple forces to achieve multiple co-governance. First of all, elderly care services are a kind of public goods, which should be led by the government to allocate resources. At the macro-level, the government conducts a scientific and reasonable top-level design, strengthens the construction of legal norms and systems, formulates complete privacy and security and ethical standards, and conducts system implementation and service quality supervision. It is also necessary to encourage, support, guide, and nurture the participation of other subjects in the process of intelligent elderly care services and actively build a complete intelligent elderly care service system. Second, it is necessary to give play to the enterprise’s characteristics of strong flexibility and a high degree of specialization in the process of service provision so as to stimulate market vitality and improve the quality and efficiency of intelligent elderly care services; the government should not only formulate sound preferential and tax policies but also constantly improve the market mechanism and provide institutional guarantee for non-government entities such as enterprises. Finally, it is necessary to emphasize the active role of social members in policy publicity and product promotion. The elderly have a single channel for obtaining information. Information dissemination among group members is an important channel for their information acquisition. This “invisible hand” must be fully utilized to carry out quality supervision and evaluation, information transmission, and promotion.

### 6.2 Improvement in the Supply of Related Facilities for Smart Elderly Care Services Based on Demand

The supply of smart elderly care services is a complete operating system. Basic network facilities, smart elderly service hardware facilities, and community service supporting facilities are closely related to the quality of smart elderly care services. The current Internet operating costs are relatively high, especially for the elderly; the network expenses are relatively high, and basic network facilities should be reduced and speeded up to ensure the application rate of the Internet. It is also necessary to strengthen the upgrading and transformation of intelligent elderly care equipment terminals, fully understand the living habits, acceptance, learning ability, and information literacy of the elderly, and explore their adaptability transformation paths so as to increase the popularity of intelligent elderly service hardware facilities. In addition, it is necessary to strengthen the training of intelligent applications for the elderly so that the elderly can master the basic application methods of intelligent elderly care equipment, increase publicity, and provide after-sales tracking services.

### 6.3 Optimization of Service Methods Based on the Platform to Achieve Dynamic Integration

The most important link in the supply of smart elderly care services is “service.” The supply of smart elderly care equipment is only the first step in service provision. The key to services is to establish a unified intelligent elderly care service platform to unify the health data and life needs of the elderly, process, and make corresponding feedback. In the survey, it is found that the elderly generally believe that to realize the transformation from traditional elderly care services to intelligent elderly care services, it is necessary to implement not only one method but also multiple methods in parallel. The demand of the elderly for smart elderly care service methods is to realize the combination of traditional services and smart services, that is, to use both online and offline service channels flexibly. To realize the expandability and extension of service items and service methods, and ensure the flexible handling of internal service and external interfaces, the basic life needs of the elderly must be fully supplied by the community, and the special life needs must be related to hospitals, governments, etc. Departments do a good job of docking to realize data sharing.

### 6.4 Promotion of the High-Quality Development of Intelligent Elderly Care Services With Talents as the Core

Smart senior care service personnel must possess a variety of professional skills such as daily nursing, health care, and intelligent applications, as well as master platform operation and service processes. From the policy level, it is necessary to formulate a sound personnel training and introduction system, introduce professional skills and service quality standards for intelligent elderly care service personnel, formulate professional training courses and teaching materials, and rely on universities for professional personnel training. At the same time, it is necessary to actively encourage members of the public to participate in smart elderly care services, broaden the source of service personnel, establish a complete talent training system, and make full use of the practical experience of such groups to enhance their ability to provide intelligent services. At the social level, it is necessary to enhance the professional identity of elderly care service personnel through methods such as increasing salary and social status and establish a complete career promotion channel. The quality of intelligent elderly care services is a key issue for the elderly, and the key to improving the quality of services is to have professional service personnel to provide efficient services. The current quality of elderly care services varies, and intelligent applications require relatively high quality of personnel. Therefore, to ensure the quality of intelligent elderly care services, it is necessary to focus on improving the quality and professionalism of service personnel.

## Data Availability

The original contributions presented in the study are included in the article/supplementary material; further inquiries can be directed to the corresponding author.
